# Expression and functional characterisation of System L amino acid transporters in the human term placenta

**DOI:** 10.1186/s12958-015-0054-8

**Published:** 2015-06-09

**Authors:** Francesca Gaccioli, Irving L. M. H. Aye, Sara Roos, Susanne Lager, Vanessa I. Ramirez, Yoshikatsu Kanai, Theresa L. Powell, Thomas Jansson

**Affiliations:** Department of Obstetrics and Gynaecology, University of Cambridge, Cambridge, UK; Division of Basic Reproductive Sciences, Department of Obstetrics and Gynaecology, University of Colorado Denver Anschutz Medical Campus, Aurora, Denver, CO USA; Institute of Biomedicine, The Sahlgrenska Academy at the University of Gothenburg, Gothenburg, Sweden; Center for Pregnancy and Newborn Research, Department of Obstetrics and Gynecology, University of Texas Health Science Center, San Antonio, TX USA; Division of Bio-System Pharmacology, Department of Pharmacology, Osaka University Graduate School of Medicine, Osaka, Japan; Section of Neonatology, Department of Pediatrics, University of Colorado Denver Anschutz Medical Campus, Aurora, Denver, CO USA

**Keywords:** Trophoblast cells, Placental nutrient transport, Leucine uptake, Maternal obesity, Pregnancy

## Abstract

**Background:**

System L transporters LAT1 (*SLC7A5*) and LAT2 (*SLC7A8*) mediate the uptake of large, neutral amino acids in the human placenta. Many System L substrates are essential amino acids, thus representing crucial nutrients for the growing fetus. Both LAT isoforms are expressed in the human placenta, but the relative contribution of LAT1 and LAT2 to placental System L transport and their subcellular localisation are not well established. Moreover, the influence of maternal body mass index (BMI) on placental System L amino acid transport is poorly understood. Therefore the aims of this study were to determine: i) the relative contribution of the LAT isoforms to System L transport activity in primary human trophoblast (PHT) cells isolated from term placenta; ii) the subcellular localisation of LAT transporters in human placenta; and iii) placental expression and activity of System L transporters in response to maternal overweight/obesity.

**Methods:**

System L mediated leucine uptake was measured in PHT cells after treatment with si-RNA targeting LAT1 and/or LAT2. The localisation of LAT isoforms was studied in isolated microvillous plasma membranes (MVM) and basal membranes (BM) by Western blot analysis. Results were confirmed by immunohistochemistry in sections of human term placenta. Expression and activity System L transporters was measured in isolated MVM from women with varying pre-pregnancy BMI.

**Results:**

Both LAT1 and LAT2 isoforms contribute to System L transport activity in primary trophoblast cells from human term placenta. LAT1 and LAT2 transporters are highly expressed in the MVM of the syncytiotrophoblast layer at term. LAT2 is also localised in the basal membrane and in endothelial cells lining the fetal capillaries. Measurements in isolated MVM vesicles indicate that System L transporter expression and activity is not influenced by maternal BMI.

**Conclusions:**

LAT1 and LAT2 are present and functional in the syncytiotrophoblast MVM, whereas LAT2 is also expressed in the BM and in the fetal capillary endothelium. In contrast to placental System A and beta amino acid transporters, MVM System L activity is unaffected by maternal overweight/obesity.

## Background

The placenta, the interface between fetal and maternal circulations, is responsible for delivering the nutrients necessary for normal fetal growth and development. Placental uptake of amino acids from the maternal circulation is mediated by transporters on the microvillous membrane (MVM) of the syncytiotrophoblast, whereas amino acid efflux to the fetal circulation is mediated by transporters on the basal plasma membrane (BM) of the syncytiotrophoblast layer. The polarised expression of amino acid transporters on the two syncytiotrophoblast plasma membranes represents the basic mechanism accounting for vectorial transfer of amino acids to the fetus [1, 2].

System L (*L*eucine preferring) is the main Na^+^-independent transporter for branched-chain (such as L-leucine) and aromatic neutral amino acids (including L-phenylalanine) [3, 4], many of which are essential. It is a heterodimer consisting of a light chain (typically *L*-type *A*mino acid *T*ransporter LAT1 (*SLC7A5*) or LAT2 (*SLC7A8*)) covalently attached to a heavy chain (CD98/4F2hc) [5]. System L transporters exchange with 1:1 stoichiometry their substrate amino acids with intracellular non-essential amino acids (e.g. L-glycine) [6], substrates of accumulative amino acid transporters, such as System A [7].

LAT1 mRNA is highly abundant in tissues containing proliferating cells or epithelial barriers such as thymus, brain and testis, while LAT2 mRNA expression is more restricted to organs that contain epithelial barriers including kidney, intestine and brain [8]. Consistent with this tissue distribution, both LAT1 and LAT2 mRNA are highly expressed in the placenta [9, 10]. The LAT1 isoform has been immunologically localised to the MVM [11] and System L activity has been reported in both the MVM and BM [12–14]. Kudo and Boyd reported that MVM System L activity is due to expression of LAT1 [15], whereas other investigators have suggested that LAT2 is the predominant isoform in the MVM [16]. In addition, transporters such as LAT4 may play a role in the efflux of certain essential amino acids across the BM [17]. Thus the subcellular localisation of LAT1 and LAT2 in the placenta and their relative contribution to System L activity in the syncytiotrophoblast remain to be fully established.

Increasing evidence suggests that placental transport function represents a link between maternal nutrient availability and fetal growth [18]. Changes in placental nutrient transport may directly contribute to abnormal fetal growth. The activity of placental amino acids transporters, including System L, is decreased in intrauterine growth restriction [19, 20]. System L transport activity has been shown to be upregulated in MVM vesicles from placentas of mothers with gestational diabetes and large for gestational age (LGA) infants [21]. Similarly, increased placental nutrient transport may represent a mechanism contributing to fetal overgrowth in pregnancies of high BMI mothers. It is however not clear if placental System L amino acid transporter expression and activity is altered in association to maternal overweight/obesity in the absence of diabetes.

In this study we investigated the contribution of LAT1 and LAT2 transporters to System L uptake in human trophoblast cells and their subcellular localisation in the term placenta. Moreover, we evaluated the expression and activity of System L transporters in MVM vesicles isolated from term placentas of women with varying pre/early pregnancy BMI.

## Methods

### Study subjects and collection of placental tissues

Healthy women with normal term pregnancies (>37 weeks of gestation) were recruited following written informed consent. Maternal BMI was calculated using the height and weight measurements from pre-pregnancy medical records or obtained at the first visit to the maternity clinic (<20 weeks). Study subjects were grouped according to their pre/early pregnancy BMI into normal (BMI < 25 kg/m^2^) and high BMI (BMI ≥ 25 kg/m^2^) groups. The latter included overweight (25 kg/m^2^ ≤ BMI < 30 kg/m^2^) and obese mothers (BMI ≥ 30 kg/m^2^). The exclusion criteria were smoking, use of illicit drugs, concurrent diseases, such as diabetes and hypertension, and development of pregnancy complications including gestational diabetes, pregnancy-induced hypertension and preeclampsia. All women included in this study had a normal glucose screening test (non-fasting, 1 h, 50 g glucose challenge). This study was approved by the Institutional Review Board at the University of Texas Health Science Center in San Antonio (HSC20100262H). Placentas were collected within 15 min of vaginal delivery or Caesarean section, coded and de-identified, and relevant medical information obtained and added to a tissue/data repository. Labor has been shown to impact placental gene expression, signalling pathways as well as the production of hormones and cytokines [22–24]. We therefore tested the influence of labor on the data in our current study, by subdividing the study subjects in those that delivered after non-laboring C-section and those delivering following labor. There was no significant difference in any of the study parameters between the groups (data not shown). Male and female samples were equally distributed between groups (Table 1).

**Table 1:** Clinical characteristics

	Normal BMI (BMI < 25 kg/m^2^)	High BMI (BMI ≥ 25 kg/m^2^)	*P*-value (*t*-test)
Patients (N)	20	24	-
Age (yrs)	28.5 ± 1.3	27.4 ± 1.0	0.51
BMI (kg/m^2^) †	21.3 ± 0.4	32.8 ± 1.1	<0.0001
Weight gain (kg)	13.7 ± 4.5	11.4 ± 4.2	0.08
Ethnicity (% Hispanic)	70 %	83 %	-
GA at delivery	39.4 ± 0.2	39.6 ± 0.2	0.45
Fetal sex (female/male)	11/9	11/13	-
Birth weight (g)	3373 ± 53	3680 ± 82	<0.01
SGA/AGA/LGA	0/20/0	0/17/7	-
Placental weight (g)	677 ± 26	798 ± 32	<0.01
Placental efficiency ††	5.1 ± 0.2	4.7 ± 0.1	0.08

Data is presented as mean ± SEM. *GA*, gestational age. † BMI, pre/early pregnancy body mass index (gestational age <20 weeks); †† Placental efficiency is calculated as the ratio between birth weight and placental weight

### Isolation and culture of primary human trophoblast cells from term placentas

Primary human trophoblast (PHT) cells were isolated from term placentas delivered by Caesarean section using a well-established method involving sequential trypsin digestion and Percoll purification [25, 26]. Cells were cultured in Dulbecco’s modified Eagle’s medium (DMEM, Sigma-Aldrich, St. Louis, MO) and Ham’s F-12 nutrient mixture (Life Technologies, Carlsbad, CA) containing 10 % of fetal bovine serum (FBS, Atlanta Biological, Atlanta, GA), 50 μg/ml gentamicin, 60 μg/ml benzyl penicillin and 100 μg/ml streptomycin (Sigma-Aldrich). Cells were plated at a density of 2.75 million in 35 mm dishes for subsequent protein analyses or 0.6 million per well in 12-well plates for uptake assays, and incubated in a 5 % CO_2_ humidified atmosphere at 37 °C. The cells were cultured for a total of 90 h with daily change of the culture media. To confirm cells differentiation, release of chorionic gonadotropin (hCG) into the cell culture media was assessed with a commercial ELISA (Immuno-Biological Laboratories, Minneapolis, MN) at 18, 66, 90 h after plating the cells. hCG concentration in the culture media was 4.3-fold (66 h) and 7-fold (90 h) higher than the concentration at 18 h after plating (*N* = 4, *P* < 0.01; data not shown). All studies were repeated in PHT cells from 3 to 4 different placentas.

### si-RNA transfection

After 18 h of culture, PHT cells were transfected with 100 nM si-RNAs targeting LAT1 and/or LAT2 transporters for 24 h using Dharmafect2 transfection reagent (Thermo Scientific, Rockford, IL), according to the manufacturer’s protocol. For both isoforms silencing efficiency was initially tested using 3 different si-RNA sequences (Sigma-Aldrich; for LAT1: SASI_Hs01_00103507, SASI_Hs01_00103508, SASI_Hs01_00103509; for LAT2: SASI_Hs01_00090978, SASI_Hs01_00090979, SASI_Hs01_00090980). Based on these pilot experiments (data not shown), SASI_Hs01_00103509 (LAT1) and SASI_Hs01_00090980 (LAT2) si-RNAs were chosen for the experiments described in this study. Equal concentration of non-targeting scrambled-siRNA (si-SCR, SIC001, Sigma-Aldrich) was used as a control. At 90 h of culture PHT cells were collected for Western blot and qRT-PCR analyses or used for transport assays. In each assay, the mean of controls (si-SCR) was assigned an arbitrary value of 1.0 and the data represents fold change from si-SCR.

### RNA Isolation and qRT-PCR analysis

Total RNA was extracted from cells using the RNeasy Mini Kit (Qiagen, Valencia, CA). Concentration and quality of the purified RNA samples were determined on a Nanodrop (Thermo Scientific). Real-time PCR was performed using SuperScript III First-Strand Synthesis SuperMix for qRT-PCR (Invitrogen, Carlsbad, CA) and SYBR green PCR master mix (Applied Biosystems, Foster City, CA), according to the manufacturer’s instructions. PCR amplification and detection was performed on Applied Biosystems Step One Plus Real-Time PCR System (Life Technologies) using the following primers: LAT1 primers: forward 5′-tccagatcgggaagggtgat-3′ and reverse 5′-ccacatccagtttggtgcc-3′; LAT2 primers: forward 5′-aggactggctgggttcctgagg-3′ and reverse 5′-gagcggctgcagcacgtagtt-3′. Succinate dehydrogenase complex, subunit A (SDHA) and TATA box binding protein (TBP) were selected as housekeeping genes [27] (SDHA primers: forward 5′-cggtccatgactctggagat-3′ and reverse 5′-agcgaagatcatggctgtct-3′; TBP primers: forward 5′-tgcacaggagccaagagtgaa-3′ and reverse 5′-cacatcacagctccccacca-3′). Amplification of a single product was confirmed by melting curve analysis. The amplified transcripts were quantified using the relative standard curve method and normalized using the geometric mean of SDHA and TBP. All samples were analysed in triplicate.

### Amino acid transport in primary human trophoblast cells

System L transport activity was determined by measuring 2-amino-2-norbornane-carboxylic acid (BCH)-inhibitable uptake of ^3^H-leucine (Leu), as previously described [26, 28]. BCH is a non-metabolizable analogue of L-leucine and it is therefore preferred in uptake experiments in cultured cells because it is not influenced by cellular metabolism. At 90 h of culture, PHT cells were washed and incubated in Tyrode’s salt solution (135 mM NaCl, 5 mM KCl, 1.8 mM CaCl_2_, 1 mM MgCl_2_, 10 mM Hepes, 5.6 mM 182 glucose; pH 7.4) with or without 1 mM BCH containing ^3^H-Leu (12.5 nM at 0.68 μCi/ml) for 8 min. After washing in cold Tyrode’s solution, cells were lysed in distilled water and radioactivity counted in a liquid scintillation counter. Protein content was determined using the Lowry method [29]. Transport activity per mg of protein was calculated and expressed as fold change from control, where the mean of the controls were assigned an arbitrary value of 1. Uptake experiments were performed in triplicate wells using cells from 3 to 4 different placentas.

### Immunohistochemistry

Villous tissue was fixed in formalin, embedded in paraffin, and cut into 5 μm sections. Immunohistochemistry was performed as described previously [30]. Briefly, the paraffin was removed by xylene and the tissue re-hydrated with ethanol. After washing with phosphate buffered saline (PBS), the slides were boiled for 10 min in citrate buffer (H-3300 Antigen Unmasking Solution; Vector Laboratories, Burlingame, CA). The immunochemical staining was performed using the Vectastain Elite ABC Kit (Rabbit IgG; Vector Laboratories). The sections were incubated with primary antibodies overnight at 4 °C in a humidified chamber. Anti-LAT1 and anti-LAT2 antibodies were produced and kindly provided by Dr Kanai (Osaka University, Japan) [31, 32]. Briefly, oligopeptides corresponding to amino acid residues 497–507 of hLAT1 [CQKLMQVVPQET] and 506–517 of hLAT2 (EEANEDMEEQQQC) were synthesized. Anti-peptide polyclonal antibodies were generated in rabbit as described by Altman et al. [33] and the antisera were affinity-purified as described by Hisano et al. [34]. Anti-LAT1 and anti-LAT2 antibodies were diluted in blocking serum (normal goat serum; LAT1 final concentration: 20 μg/ml; LAT2 final concentration: 40 μg/ml). A commercially available anti-LAT1 antibody was also used (Capralogics, Hardwick, MA) and diluted in blocking serum at 20 and 40 μg/ml final concentrations. Non-specific staining was detected by incubating the sections in blocking serum without the primary antibody (negative controls) or in the presence of Rabbit IgG (20 and 40 μg/ml). Subsequently, sections were incubated in secondary antibodies for 1 h at room temperature. Staining was visualised with DAB Peroxidase Substrate Kit (Vector Laboratories). Sections were counterstained with Harris modified Hematoxylin (Fisher Scientific, Pittsburgh, PA) and visualised using a Leica DMRB microscope and 40X N PLAN objective (Leica, Wetzlar, Germany). Images were acquired with an Olympus DP71 digital camera (Olympus, Hamburg, Germany).

### Preparation of syncytiotrophoblast microvillous (MVM) and basal plasma membranes (BM)

Placental plasma membranes were prepared from a total of 44 placentas as previously described [35]. Briefly, approximately 100 g of villous tissue was homogenized using a Polytron (15,000 rpm, 2 min) on ice-cold buffer D (250 mM sucrose, 10 mM hepes, pH 7.4 with protease and phosphatase inhibitors), snap-frozen in liquid nitrogen and stored at −80 °C until use. After further processing of the placental homogenates through sequential Mg^2+^ precipitation and differential centrifugations, the final pellets were resuspended in buffer D (250 mmol/L sucrose, 10 mmol/L hepes, pH 7.4 at +4 °C; final protein concentration: 5–20 mg/ml), aliquoted, and stored at −80 °C until use. The protein concentration in MVM and BM vesicles was determined using the Pierce BCA Protein Assay kit (Thermo Scientific). As described previously [36], the enrichment of BM was determined using the protein expression of the iron transporter ferroportin-1 (*SLC40A1*), which is almost exclusively expressed on the BM to facilitate unidirectional iron transport from mother to fetus. The mean enrichment of ferroportin expression in BM was 15.7 ± 2.5. MVM enrichments were assessed using standard activity assays for alkaline phosphatase [37]. Only MVM preparations with an alkaline phosphatase activity enrichment of >10-fold compared to placental homogenates were included in the study. Enrichment of alkaline phosphatase activity in the MVM samples and enrichment of ferroportin in the BM samples were not significantly different in placentas obtained from normal and high BMI women.

### System L amino acid transport activity in MVM vesicles

The activity of System L amino acid transporters was determined in a subset of MVM vesicles (*N* = 43) as described previously [38]. MVM vesicles were preloaded by incubation in 300 mM mannitol and 10 mM HEPES-Tris, pH 7.4, overnight at 4 °C. Subsequently, vesicles were pelleted and resuspended in a small volume of the same buffer to a final protein concentration of 5–10 mg/ml. After warming the samples to 37 °C, 30 μl of vesicles were rapidly mixed (1:2), with the appropriate incubation buffer including ^3^H-L-leucine (0.375 μM). After 10 s, the uptake of radio-labeled leucine was terminated by addition of 2 ml ice-cold PBS. Subsequently, vesicles were rapidly separated from the substrate medium by filtration on mixed ester filters (0.45 μm pore size; Millipore Corporation, Billerica, MA) and washed with 4 × 2 ml ice-cold PBS. Filters were dissolved in 2 ml liquid scintillation fluid and counted. In leucine transport experiments the non-mediated flux was determined in the presence of 30 mM unlabeled L-leucine, and mediated uptake was calculated by subtracting non-mediated transport from total uptake. Protein content of the vesicles was determined by Pierce BCA Protein Assay kit (Thermo Scientific) and uptakes were expressed as picomoles per milligram of protein. In all uptake experiments, each condition was studied in triplicate.

### Western blot analysis

Cellular and placental membrane protein expression of System L isoforms was determined by Western blot analysis, carried out as previously described [26, 39]. Briefly, cell protein lysates were prepared in RIPA buffer and protease inhibitors and phosphatase inhibitor cocktail 1 and 2 (1:100, Sigma Aldrich) were added to cell lysates and MVM and BM vesicles. Proteins were separated by SDS-PAGE electrophoresis using Mini-Protean TGX precast gels (Bio-Rad, Hercules, CA) and transferred to PVDF membranes (35 V constant, overnight at 4 °C). Membranes were stained with Amido Black staining solution for total proteins (Sigma-Aldrich) according to the manufacturer’s instructions and then blocked in 5 % non-fat milk in Tris-buffered saline containing 0.1 % Tween (TBS-Tween) for 1 h at room temperature. After brief washing in TBS-Tween, membranes were incubated overnight with antibodies targeting LAT1 and LAT2 (2 μg/ml) or beta-actin (0.2 μg/ml; Sigma-Aldrich). After washing, the membranes were incubated with the appropriate peroxidase conjugated secondary antibody and visualised using ECL detection solution (Thermo Scientific). Densitometry analysis was performed with ImageJ software (National Institutes of Health, USA). Target protein expression was normalized to beta-actin expression.

### Data presentation and statistical analysis

Data are presented as mean ± SEM. Statistical significance was determined by Student two-tailed *t*-test or one-way ANOVA followed by Dunnett’s *post hoc* test. Pearson’s correlation was used to assess the association between transport activity and birth weight. Statistical analysis was performed using GraphPad Prism 5 software (La Jolla, CA). *P* < 0.05 was considered significant.

## Results

### Both LAT1 and LAT2 isoforms contribute to System L transport activity in term PHT cells

**Fig. 1:**
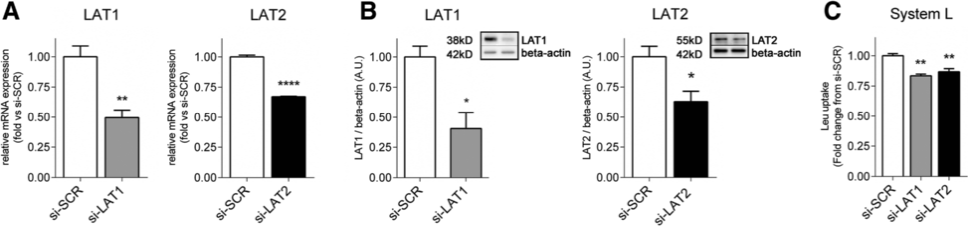
Decrease of System L activity after silencing of LAT1 or LAT2 transporters. PHT cells were transfected with non-targeting scramble-siRNA (*si-SCR*), LAT1-siRNA (*si-LAT1*) or LAT2-siRNA (*si-LAT2*). **a** mRNA expression of LAT1 and LAT2, normalized against the housekeeping genes SDHA and TBP. **b** Representative immunoblots of LAT1, LAT2 and the corresponding beta-actin following si-RNA treatments. Histograms illustrate relative protein expression. **c** System L amino acid transport activity following si-RNA treatments. Mean of controls was assigned an arbitrary value of 1 and data represents fold change from si-SCR; mean + SEM, *N* = 3. Student *t*-test (**a** and **b**) or one-way ANOVA (**c**), *P < 0.05, **P < 0.01, ***P < 0.001, ****P < 0.0001 *vs* control (*si-SCR*)

**Fig. 2:**
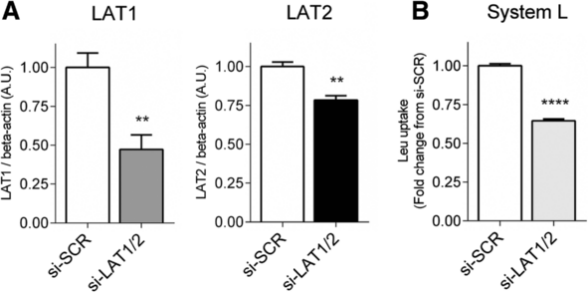
Silencing of both LAT isoforms additively reduces System L transport activity. PHT cells were transfected with non-targeting scramble-siRNA (*si-SCR*) or LAT1-siRNA and LAT2-siRNA (*si-LAT1/2*). **a** Histograms illustrate relative protein expression of LAT1 and LAT2 following si-RNA treatments, *N* = 3. **b** System L amino acid transport measured after si-RNA treatments, *N* = 4. Mean of controls was assigned an arbitrary value of 1.0 and data represents fold change from si-SCR; mean + SEM. Student *t*-test, **P < 0.01, ****P < 0.0001 *vs* control (*si-SCR*)

Transfection of term PHT cells with LAT1- or LAT2-specific si-RNAs (si-LAT1 or si-LAT2) decreased their mRNA expression by 50 % (*P* < 0.01, *N* = 3) and 33 % (*P* < 0.0001, *N* = 3), respectively, compared to scramble (si-SCR) transfected cells (Fig. 1a). Consistent with the mRNA levels, si-RNA silencing reduced LAT1 and LAT2 protein expression by 59 and 37 % (*P* < 0.05, *N* = 3), respectively, compared to the control group (Fig. 1b). To determine the effect of LAT1 and LAT2 silencing on System L activity, we measured BCH-inhibitable uptake of ^3^H-leucine in term PHT cells following si-RNA treatments. Transfection with LAT1-siRNA decreased System L activity by 16 % (*P* < 0.01, *N* = 3; Fig. 1c). Similarly, LAT2 silencing resulted in a 13 % decrease in System L activity (*P* < 0.01, *N* = 3; Fig. 1c). In cells transfected with si-RNA targeting both LAT1 and LAT2, silencing efficiency was similar to single transfections because LAT1 protein expression decreased by 53 % (*p* < 0.01, *N* = 3) and LAT2 expression by 22 % (*p* < 0.01, *N* = 3; Fig. 2a). Silencing both LAT1 and LAT2 produced an additive effect on System L transport resulting in 35 % decrease in activity (*p* < 0.001, *N* = 4; Fig. 2b).

### Localisation of LAT isoforms in the human term placenta

**Fig. 3:**
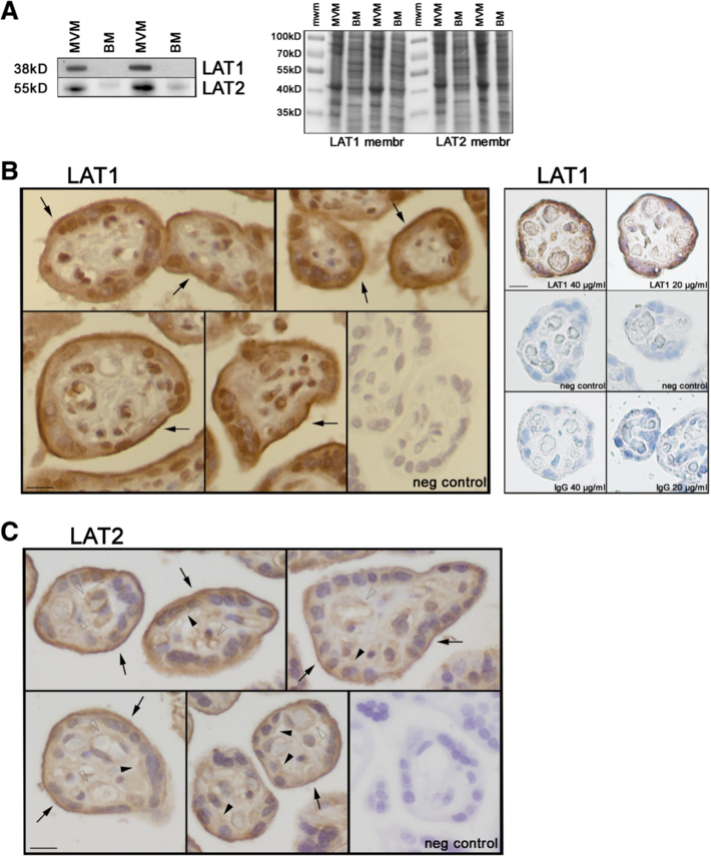
Localisation of LAT transporters in term placenta. **a** MVM and BM expression of LAT1 and LAT2 analysed by Western blot (*left*) and total protein staining of the two corresponding PVDF membranes (*right*). Equal amounts of proteins were loaded in each lane (20 μg/lane). *mwm* molecular weight marker. **b-c** Representative immunohistochemistry images of LAT1 and LAT2 in human term placenta (*N* = 3 for each condition). LAT1 localisation was assessed using two different antibodies (panel **b**, *left*: antibody provided by Dr Kanai at 20 μg/ml final concentration; panel **b**, *right*: antibody by Capralogics at 40 μg/ml and 20 μg/ml final concentrations). Negative controls and Rabbit IgG staining (40 μg/ml and 20 μg/ml final concentrations) are shown. Nuclei stained with hematoxylin. MVM (*black arrow*), BM (*black arrowhead*), capillary endothelium (*empty arrowhead*). *Scale bar* 20 μm

Equal amounts of matching MVM and BM samples were analysed by Western blot in order to study the relative distribution of the LAT transporter isoforms in the syncytial plasma membranes at term (Fig. 3a). LAT1 protein expression was highly expressed in the MVM, but it could not be detected in the BM samples. While predominantly expressed in the MVM, LAT2 isoform was also detected in the BM, albeit at a much lower expression level (Fig. 3a). Densitometry analysis was not affected by non-homogeneous protein transfer, as shown by total proteins staining of the two Western blot membranes (Fig. 3a), and demonstrated that LAT2 expression was 5.3-fold higher in the MVM as compared to BM. Placental localisation of LAT isoforms was further assessed by immunohistochemistry of term placentas. Consistent with the Western blot results, immunohistochemical staining for both LAT1 and LAT2 was predominantly detected in the maternal-facing MVM of terminal villi (Fig. 3b-c; MVM: black arrow). A diffuse staining for LAT1 was also present in the cytoplasm of the syncytiotrophoblast layer (Fig. 3b). Although LAT2 staining was prominently in the MVM, it could also be detected in the BM (Fig. 3c; BM: black arrowhead), in agreement with the Western blot data. Staining for LAT2 was also present in the endothelium surrounding the fetal capillaries (Fig. 3c; endothelium: empty arrowhead).

### Expression and activity of System L amino acid transporters in isolated MVM vesicles is not affected by maternal BMI

**Fig. 4:**
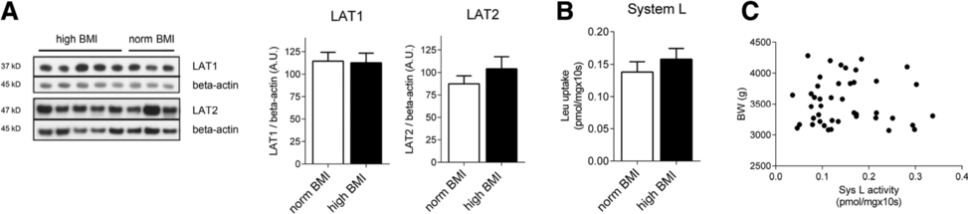
Protein expression and activity of System L amino acid transporters in MVM from women with normal or high pre/early pregnancy BMI. **a** Representative Western blots showing MVM expression of LAT1 and LAT2 transporters in mothers with normal and high pre-pregnancy BMI. *Bar graphs* represent the summary of data; mean + SEM. LAT1: *N* = 6, normal BMI; *N* = 9, high BMI. LAT2: *N* = 17, normal BMI; *N* = 21, high BMI. **b** System L transport activity was determined in isolated MVM vesicles; mean + SEM. *N* = 19, normal BMI; *N* = 24, high BMI. **c** Correlation between placental MVM System L activity and birth weight (*N* = 43)

Table 1 summarizes selected clinical data for the study subjects grouped according to maternal pre/early pregnancy BMI. Per study design, maternal BMI is significantly higher in the high BMI compared to the normal BMI group; as expected, maternal weight gain tended to be lower in overweight/obese mothers (*P* = 0.08). Consistent with studies on larger populations [40], birth weights and placental weights were significantly higher in the high BMI compared to the normal BMI group (Table 1). In light of the results on the localisation of LAT isoforms (Fig. 3), we studied the expression of System L transporters LAT1 and LAT2 only in isolated MVM vesicles from placentas of women with normal and high BMI. There were no significant differences in LAT1 or LAT2 MVM expression between the two groups (Fig. 4a). In the same MVM-vesicle preparations System L transport activity was not affected by maternal overweight/obesity (Fig. 4b), consistent with the protein expression data. Neither LAT1 and LAT2 expression nor System L activity were different between groups after dividing the high BMI mothers into overweight and obese (data not shown). System L activity and LAT isoforms expression in MVM vesicles did not correlate with maternal weight gain (data not shown). In addition, there was no correlation between System L activity and birth weight (Fig. 4c; *P* = 0.88).

## Discussion

This is the first time LAT1 and LAT2 mediated transport has been studied in primary trophoblast cells using gene targeting approaches. Consistent with previous competitive inhibition studies [41], we report that both LAT1 and LAT2 isoforms contribute to System L leucine uptake in primary trophoblast cells from human term placenta. We used si-RNA transfections targeting LAT1 and/or LAT2 isoforms to evaluate the effect of these treatments on System L amino acid transport activity. After specifically targeting LAT1 or LAT2, the decrease in System L transport activity was, in general, proportional to the decreases in LAT1 and LAT2 protein expression following si-RNA treatments. Moreover, silencing both LAT1 and LAT2 transporters resulted in an additive reduction of leucine uptake. These results suggest that both transporters independently contribute to trophoblast System L activity. Isolated human primary trophoblast cells syncytialize spontaneously in culture. During syncytialisation, trophoblast cells become polarized and develop abundant and regular microvilli on the cell surface facing the culture media [42–44]. This is the cell surface that is freely accessible in vitro during transport activity measurements and it is very likely that the measured uptake can be accounted for transport across the MVM of the syncytiotrophoblast layer.

Using Western blot and immunohistochemistry we also show the localisation of LAT1 and LAT2 in the human term placenta. With both methods we demonstrated that LAT1 is mainly localised in the syncytiotrophoblast apical/microvillous plasma membrane, confirming the immunohistochemistry results from Okamoto et al. [11]. These data are in agreement with the model that LAT1 is mainly involved in the uptake of large neutral amino acids at the maternal side of the syncytiotrophoblast layer [7]. To the best of our knowledge, the localisation of LAT2 in the placenta has so far been explored only by functional studies. Whereas some studies indicate that LAT2 is polarized to the basal membrane of the syncytiotrophoblast layer [15], other investigators have suggested that LAT2 is the predominant isoform in the MVM [16]. Our immunohistochemistry and Western blot data demonstrate that LAT2 is highly expressed in the MVM in vivo and that this isoform contributes to apical leucine transport in primary cytotrophoblast cells in culture. The mechanisms underlying the efflux of amino acids through the BM is still largely unknown, but System L has been indicated as a strong candidate contributing to this process. Our results on the localisation of LAT2 in the BM membranes and in the epithelium of the fetal capillaries support the notion that this exchanger could mediate the efflux of amino acids from the syncytiotrophoblast layer (in analogy to its function in the absorptive epithelia of the digestive tract [8]) and their transfer to the fetal circulation. Experimental data by Cleal and co-workers in the isolated perfused human placental cotyledon indicate that one System L isoform mediates L-leucine exchange in the fetal-facing BM [17]. However, the same investigators suggested that LAT1, rather than LAT2, is the main BM exchanger and that non-exchange mechanisms also contribute to efflux of leucine across the BM [17, 45]. This apparent discrepancy could be explained by the fact that LAT2 appears to mediate facilitated transport of neutral amino acids (in addition to function as a true exchanger [46, 47]) and LAT2 may therefore also contribute to BM amino acid transport by non-exchange mechanisms.

The link between maternal nutrition, placental nutrient transport function and fetal growth has been studied in various animal models of maternal undernutrition and diabetes, which are often associated with restricted and accelerated fetal growth, respectively [18]. The studies discussed above support the hypothesis that placental transport capacity is regulated by maternal nutritional cues and fetal signals in order to balance fetal demand with the ability of the mother to support pregnancy and the growing fetus [18, 48]. Consistent with this model, the activity and/or expression of amino acid transporters System A and System L was previously reported to be decreased in placentas of intrauterine growth restricted infants [19, 49–52] and increased with fetal overgrowth associated with maternal diabetes [21, 53]. Whether fetal overgrowth could be explained by altered placental nutrient transport capacity is less clear in pregnancies of high BMI women without diabetes. Nevertheless, the endocrine and metabolic perturbations induced by maternal obesity emerge as important factors regulating placental nutrient transport function [36, 38, 54–58]. For example, placental mRNA of Glucose Transporter (GLUT)-4 has been shown to be significantly decreased in obese compared to normal BMI patients [57], while BM protein expression of GLUT-1, but not glucose transport activity, was positively correlated with birth weight in women with varying pre-pregnancy BMI [36]. Dube et al. [58] reported altered fatty acids transport and metabolism in the placenta of obese women. With respect to placental amino acid transport, decreased System A activity and lower expression of the System A isoform SNAT4 was reported in placental villous fragments isolated from term placentas of obese Hispanic women giving birth to AGA babies [55]. Possibly due to differences in the ethnicity of the study populations and in fetal outcome, System A activity was increased in MVM isolated from placentas of obese women giving birth to large babies, in a cohort of 23 Swedish women [38]. In the same study the expression of the SNAT2 transporter was positively correlated with maternal BMI and birth weight. Consistent with our current results, System L activity and transporters’ expression were not altered in response to varying maternal BMI in the Swedish cohort, suggesting that System A amino acid transporters may be more sensitive to the metabolic and endocrine changes in maternal obesity. As System A provides the substrates for System L exchange mechanism, it is possible that an increase in placental System A activity in pregnancies complicated by maternal overweight/obesity could enhance System L efficiency in vivo. In this report we established that in our study population the MVM activity of System L amino acid transporters is not modulated by maternal overweight/obesity and does not correlate with birth weight. Notably, by excluding study subjects with diabetes and hypertension, we attempted to isolate maternal overweight/obesity from these two common comorbidities [59–61]. Pre-existing and gestational maternal diabetes have been shown to alter the effect of maternal obesity on pregnancy outcomes [62–65], maternal metabolism [66] and placental function [67]. Our data indicate that high maternal BMI *per se* does not modify the expression and activity of System L transporters in absence of other aggravating conditions, such as diabetes and hypertension. Nevertheless, the increased placental size in the high BMI group suggests that a larger placental exchange surface area could contribute to increased fetal nutrient supply and birth weight in this group. It also possible that elevated maternal nutrient levels and/or upregulation of placental nutrient transporters not examined in our current study could enhance placental nutrient transfer and promote fetal growth in some obese women.

## Conclusions

In conclusion, we have demonstrated that both LAT1 and LAT2 contribute to leucine uptake in cultured primary human trophoblast cells. While both LAT1 and LAT2 were predominantly localised to the MVM of human term placentas, LAT2 was also present in the BM and the endothelial cells lining the fetal capillaries. The expression and the activity of placental System L transporters in MVM vesicles were not influenced by maternal overweight/obesity in the absence of diabetes.

## Abbreviations

BCH: 2-amino-2-norbornane-carboxylic acid
BM: Basal membrane
BMI: Body mass index
DMEM: Dulbecco’s modified Eagle’s medium
FBS: Fetal bovine serum
LAT: L-type Amino acid Transporter
Leu: Leucine
MVM: Microvillous plasma membrane
PBS: Phosphate buffered saline
PHT cells: Primary human trophoblast cells
TBS-Tween: Tris-buffered saline containing Tween
